# Parent Intention to Enroll in an Online Intervention to Enhance Health Behavior Change among Youth Treated with Psychotropic Medication Who Are Overweight or Obese: An Elicitation Study

**DOI:** 10.3390/ijerph19138057

**Published:** 2022-06-30

**Authors:** Kathryn A. Richardson, Christine L. McKibbin, Barbara S. Dabrowski, Elizabeth L. A. Punke, Cynthia M. Hartung

**Affiliations:** Department of Psychology, University of Wyoming, Laramie, WY 82072, USA; kricha21@uwyo.edu (K.A.R.); bdabrows@uwyo.edu (B.S.D.); epunke@uwyo.edu (E.L.A.P.); chartung@uwyo.edu (C.M.H.)

**Keywords:** youth, overweight and obesity, psychotropic medication, online intervention, theory of planned behavior, qualitative study

## Abstract

Youth who are prescribed psychotropic medication are disproportionally affected by overweight/obesity (OW/OB), yet few interventions have been tailored to their needs. To develop new interventions, it is important to address the needs, preferences, and intentions of target users. Qualitative methods within the theory of planned behavior (TPB) framework were used in this study to identify salient beliefs which may influence attitudes associated with parents’ intentions to participate in a future online intervention designed to develop behavioral health coaching skills among parents and guardians. Twenty parents and guardians of youth with OW/OB who were taking psychotropic medications, and were eligible for the study, were recruited through TurkPrime. Parents and guardians identified key salient beliefs consistent with the theory of planned behavior including behavioral beliefs (e.g., access and convenience), normative beliefs (e.g., family), and control beliefs (e.g., cost) that may influence their decision to enroll in a future, parent-oriented intervention. The results of this study suggest important salient beliefs which may be included in future research, as well as specific preferences which may be used to guide the development of a future intervention. Future work should focus on the creation of a salient belief quantitative measure and assess the relationships of these beliefs to attitudinal constructs and behaviors.

## 1. Introduction

Overweight and obesity (OW/OB) among youth is a growing epidemic worldwide and in the United States (US). Globally, the number of obese youths aged 5–19 years has risen more than tenfold, from 11 million in 1975 to 124 million in 2016. An additional 213 million were overweight in 2016 [1]. The prevalence of obesity among children and adolescents has increased worldwide from 4% in 1975 to over 18% in 2016 [2]. Among the countries examined, the US is ranks high for the prevalence of youth with overweight and obesity. In fact, data retrieved from the 2015–2016 National Health and Nutrition Examination Survey reported that 16.6% of youths aged 2–19 living in the US met criteria for overweight and 18.5% met criteria for obesity [3]. Importantly, OW/OB in childhood and adolescence increases the risk of OW/OB persisting into adulthood [4] and is associated with serious health consequences such as periodontal diseases [5], asthma [6], cardiovascular disease [7], metabolic syndrome, type 2 diabetes, hypertension, dyslipidemia, and obstructive sleep apnea [8]. A recent narrative review by Chao and colleagues [9] suggested that youth with mental health disorders (MHD; e.g., bipolar disorder, schizophrenia, depression, anxiety) are disproportionally affected by OW/OB and related diseases. Considering that an estimated 10–20% of youth worldwide have a mental health disorder [10], a substantial proportion of youth with MHD may experience OW/OB.

Multiple correlates have been identified among youth who experience both OW/OB and MHD. Chao and colleagues [9] indicated in their review that youth with MHD have poorer dietary and physical activity behaviors compared to youth from the general population. Specifically, youth with depression had a higher calorie intake and consumption of sweets [11], decreased physical activity, increased screen time [12], and increased sedentary activity [13] when compared to controls. Similarly, youth with bipolar disorder also self-reported nutrition and dietary excess related to stress-induced eating [14]. Additionally, stigma related to OW/OB and mental health may worsen both mental health and OW/OB outcomes for these youth [9]. 

Psychotropic medications (e.g., antipsychotics, mood stabilizers, antidepressants), which have United States Food and Drug Administration-approved indications for youth with MHD, may also exacerbate weight gain and cardiometabolic risk [9,15]. For example, Correll and colleagues [16] found that effects of psychotropic medications reported in children and adolescents led to a greater magnitude of weight gain following exposure to antipsychotic medication. Additional studies found that children and adolescents who use antidepressants appear to have an elevated risk for type 2 diabetes mellitus (DM) [17,18]. Relatedly, children who are prescribed second-generation antipsychotics (SGAs) were shown to have a 2–3 times higher risk of developing type 2 DM compared with SGA-naive children [19]. Recent evidence has also suggested that, among antipsychotic-treated youth, concomitant SSRI/SNRI use is associated with an even higher risk of type 2 DM, which markedly intensifies with increasing duration of SSRI/SNRI use and cumulative SSRI/SNRI dose [18,20]. Yet, given that it may not be feasible for these youth to discontinue or switch their psychotropic medications, health interventions may be necessary to help mitigate these risks.

Numerous studies and systematic reviews have demonstrated the short-term value of lifestyle interventions to address OW/OB and health behaviors among typical youth, as well as the importance of family involvement in interventions [21]. Likewise, a smaller number of interventions have been developed to address the unique needs of OW/OB among youth, young adults, and adults who are living with mental health conditions [22,23]. Yet, few of these interventions have included a family component [24] and most, if not all, have been designed to be delivered in face-to-face formats. Face-to-face delivery may limit access to families with barriers to intervention engagement such as transportation difficulties, time constraints, and parenting stress [25]. 

More recently, internet-based interventions, or e-health interventions, have been developed to increase access to health promotion programming among parents of youth with OW/OB in the general population. Hammersley and colleagues [26] conducted a systematic review of seven studies involving parent and child or adolescent dyads (i.e., three obesity prevention trials and four obesity treatment trials). They found that, in four of the seven studies that reported on dietary outcomes, a significant change occurred in at least one dietary measure. They also found that, among the six studies addressing physical activity outcomes, only one study showed improvement in physical activity behavior. None of the studies found a significant difference between the intervention and control groups on a measure of body mass index. The authors noted that the quality of the studies was not high and called for researchers to transform the effective components of face-to-face interventions into internet-based programs. It is also important to recognize that none of the programs reviewed were developed specifically for parents of youth with MHD and OW/OB. Therefore, additional work is needed to design and create interventions for this population, particularly those parents of youth with MHD and OW/OB who are taking psychotropic medications. 

Prior to developing and testing an online program to address the needs of parents of youth with OW/OB who take psychotropic medications, a thorough understanding of factors influencing parent intention to enroll in such a program is needed. Obtaining parent perspectives early in intervention development may assist in the design of a program that would be relevant for and appeal to the target population of parents and caregivers who support youth with MHD, thereby enhancing efficiency in intervention development and minimizing research waste [27]. 

The theory of planned behavior (TPB) is a theoretical framework that is widely used to predict and explain the intentions of an individual to engage in a targeted behavior within a specific context [28,29]. This framework evolved from the theory of reasoned action which states that intentions are the best predictor of behavior. According to the TPB, the prediction of behavioral intentions are the product of three *direct attitudes*: attitude toward the behavior, subjective norm, and perceived behavioral control. *Attitude toward the behavior* refers to how one thinks and feels about a behavior and reflects one’s values and expectations of a behavior. *Subjective norm* refers to the support given by significant others such as family, friends, or others in the support network such as a mental or physical health provider. Finally, *perceived behavioral control* refers to the extent to which one believes they are capable and confident in their ability to execute the targeted behavior and overcome potential barriers. Taken together, the TPB posits that when an individual perceives an activity as enjoyable and with good benefits, where support and encouragement from others are available and/or where others are engaging in similar behaviors, and when one believes in one’s own capability of meeting the demands of the task, one will form stronger intentions, and be more likely to engage in a particular activity.

*Salient beliefs* (i.e., behavioral beliefs, normative beliefs, control beliefs), which precede and are related to each of the TPB attitudinal constructs, are those beliefs that come to mind when one is asked key open-ended questions (e.g., “What do you believe are the advantages of enrolling in an online health and wellness intervention in order to help your child or adolescent with a mental health disorder?”). These are also known as accessible beliefs and are necessary for explaining intentions and subsequent engagement in a specific behavior. Importantly, information about specific salient beliefs can provide valuable information required to produce a change in attitudes [28]. The theory holds that an individual’s salient beliefs determine direct attitudes and that these beliefs must be identified prior to constructing a measure of attitudes, which is needed to predict behavioral intentions. 

The TPB is a valuable framework for understanding health behaviors. For example, TPB has been used to understand the intentions of community mental health providers to engage in the provision of structured weight-loss interventions to youth with serious emotional disturbance (i.e., MHD) who are served in the community mental health setting [30,31]. This theory has also been used to understand factors influencing the intentions of parents of youth (i.e., primary school age) with OW/OB to enroll in a weight management program [32]. Importantly, the TPB has been used in formative intervention development research. Specifically, the TPB has been employed in intervening health campaigns designed to persuade people either to not participate in a dangerous behavior or to start participating in a healthy behavior. For example, the theory has served as a foundation to address parent engagement in behaviors such as engagement in preventative parenting programs to reduce child mental health disparities [33] and the risk of adverse child outcomes [34]. TPB has also been employed in studies to develop health messages that promote health behavior changes such as increased fruit and vegetable consumption, increased physical activity, and reduced alcohol consumption [35,36].

The Technology Acceptance Model (TAM) is, similar to the TPB, derived from the theory of reasoned action and has also been widely adopted to study the intentions and behavior of using technologies. However, the TPB is argued to provide more useful information for intervention development than the TAM [37,38]. For example, the TAM focuses on the adoption of information technology for the workplace and is centered on constructs such as perceived usefulness (i.e., the degree to which a person believes that using a particular system would enhance his or her job performance) and the ease of use of a technology (i.e., the degree to which a person believes that using a particular system would be free of effort) [39]. In this case, the innovation is already developed and users provide feedback on perceived usefulness and the ease of use of the technology. Some overlaps between TPB and TAM exist; in fact, more recent TAM models have included other diverse needs such as social influence processes (e.g., subjective norms) as antecedents to these two key constructs [40]. Other research has examined blended models, including both TAM and TPB. However, blending the use of the two theories in explaining the uptake of technologies is controversial [41], and individual theories have been found to offer more convincing results than integrated theories [42]. Therefore, the TPB was selected for this study because the focus of this study is on the development of online interventions rather than on perceived ease of use, acceptance, and social influence on the intention to use or actually use an existing tool.

The primary goal of this study is to elicit salient beliefs which can then be used to inform the development of attitudinal measures included in the TPB. This study represents the first step in the study of factors that would influence parents’ intentions to enroll in online interventions for developing parental behavioral health coaching skills. The authors of the TPB recommend that researchers who use this theory to investigate the determinants of a given behavior should first conduct an elicitation study to identify the modal salient beliefs in the target population. Therefore, preliminary research is needed to elicit salient beliefs related to parents’ intentions to enroll in online skills development interventions. The intervention will be described in general terms as the actual online program is not yet developed. Modal salient beliefs may be identified by conducting an elicitation study in which responses to open-ended questions are recorded and summarized across participants by way of content analysis. In addition to using belief elicitation to inform the assessment of attitudinal constructs, in the TPB, a secondary goal of the study is to elicit the perspectives of the target population (i.e., parents and guardians) on the design features (e.g., messages, modalities, platforms) that may be important to consider in future online intervention development. The elicitation of salient beliefs may provide useful information to support intervention development.

## 2. Materials and Methods

### 2.1. Participants

This study was approved by the University of Wyoming’s Institutional Review Board (IRB) and was developed and conducted between November 2019 and March 2020. Participants were recruited by convenience sampling through TurkPrime, an online research platform that recruits participants with select demographics (e.g., parents and guardians) from Amazon’s Mechanical Turk (MTurk) [43]. MTurk has been validated in research on psychopathology as a time-effective means of collecting high-quality data in clinical populations [44]. Participants self-selected to participate in the study. All measures were completed through Qualtrics [45]. A total of 97 participants successfully consented to the study, completed phase one screening, demonstrated adequate attention (i.e., passed at least 2 of 3 attention checks), produced coherent responses, and were compensated USD 1.50 for their time. The study took approximately 15–20 min to complete. Of those who were compensated, 20 parents or guardians met eligibility criteria (i.e., age 18 or older, primary caregiver of a youth aged 11–17 who takes psychotropic medication and is OW/OB, residing with their youth in the US, English speaking, able to provide informed consent, and demonstrated adequate understanding of the prompt). OW/OB status and BMI were calculated based on the parent-reported biological sex, age, height, and weight of the youth. Demographic and clinical history data were collected for parent and guardian participants and their respective youths.

As shown in Table 1, the demographic data for parents and guardians suggest a majority were cisgender female, White, and non-Hispanic/Latinx. A majority were also married, had a bachelor’s degree, were employed full-time, and had a net income of USD 50,001 and above. Respondents were primarily born in the US. Most of the sample comprised parents rather than guardians (*n* = 16, 80%). Parents and guardians ranged in age from 29 to 60 years, averaging 42.5 years old. Clinical history for parents and guardians suggested the average BMI for parents and guardians was 31.4 (SD = 10.6), with the majority (*n* = 13, 65%) qualifying for OW/OB status. Despite the high calculated prevalence of OW/OB in this sample, only a minority (*n* = 6, 30%) reported receiving a formal diagnosis of OW/OB. Additionally, over one-half (*n* = 11, 55%) of parents and guardians reported having one or more mental health diagnoses themselves, with the most common mental health diagnoses including anxiety and depression. Over one-half of parents and guardians reported taking a psychotropic medication, with the most frequently prescribed psychotropic medication category being antidepressants. Just over half of the sample also reported receiving one or more physical health diagnoses themselves, with the most common physical health diagnoses including OW/OB and high blood pressure. The entire sample reported using health or mental health services, with the most common services used including primary care and community mental health. 

Parents and guardians reported on one identified youth with a mental health disorder. Most of these youths were cisgender male (*n* = 13, 65%), White (*n* = 16, 80%), and non-Hispanic/Latinx (*n* = 18, 90%). Youths ranged in age from 11 to 17 years (M = 13.6, SD = 2.1) and ranged in grade level from 4th- to 12th-grade education. All youth were overweight or obese (i.e., female BMI z-score, *M* = 1.70, *SD* = 0.76; male BMI z-score, *M* = 2.00, *SD* = 0.82). The majority of female (*n* = 4, 57.2%) and male (*n* = 9, 69.3%) participants were classified as obese. The remainder were classified as overweight. Of these youths, only 10% (*n* = 2) received a formal diagnosis of OW/OB. The entire sample of youths had at least one mental health diagnosis, with the majority (*n* = 15, 75%) of parents and guardians reporting more than one mental health diagnosis in their youth; the most common diagnoses in youths included anxiety (*n* = 10, 50%) and autism spectrum disorder (ASD; *n* = 9, 45%). All youths were reportedly prescribed a psychotropic medication, with the most frequently used psychotropic category being antidepressants (*n* = 11, 55%). The entire sample of youths were reported to use services, with the most common services used including community mental health (*n* = 8, 40%) and school-based services (*n* = 8, 40%). Nearly all youths were reported to have had a physical examination in the past year (*n* = 19, 95%).

### 2.2. Measures

#### 2.2.1. Screening Phase-One Questionnaire

The Screening Phase-One Questionnaire comprised a series of three questions designed to identify the caregiver status of the target population (i.e., the caregiver of a youth with a mental health disorder) with the inclusion of decoy questions about other populations (i.e., caregivers of youths with intellectual disabilities; caregivers of youths with physical disabilities) designed to obscure eligibility criteria. Participants who responded “yes” to any of the three questions about caregiving were directed to six follow-up questions (e.g., Have you noticed that this youth is overweight? Are you the primary caregiver of this youth?).

#### 2.2.2. Sociodemographic Questionnaire

A 19-item multiple-choice and short-answer demographics form was used to collect basic information about personal characteristics (e.g., age, biological sex, gender identity, height, weight) and the clinical history of the participants (e.g., diagnoses, medications).

#### 2.2.3. Youth Clinical History Questionnaire

An 18-item multiple-choice and short-answer youth clinical history questionnaire was used to collect basic information about personal characteristics (e.g., age, biological sex, gender identity, height, weight) and the clinical history of the youth (e.g., diagnoses, services used). Reported weight and height were used to calculate an estimated body mass index (BMI). BMI was recoded into a BMI z-score standardized for age and gender based on World Health Organization (WHO) simplified field tables. WHO cut-off scores were referenced for determining overweight and obesity status [46].

#### 2.2.4. Salient Belief Elicitation Questionnaire

A 12-item Salient Belief Elicitation Questionnaire, based on established TPB guidelines [29], was used for this study. As shown in Table 2, participants were instructed to consider the possibility of enrolling in a general online health and wellness intervention to teach parents to serve as behavioral health coaches for their children with a mental health disorder. Specific content of the intervention was not presented, but general intervention methods (e.g., didactic content, peer support) were shown. Eleven open-ended questions reflected three belief domains (i.e., normative beliefs, control beliefs, and behavioral beliefs). Five open-ended questions assessed behavioral beliefs. Two questions assessed the instrumental component of behavioral beliefs (i.e., advantages and disadvantages of performing the behavior) as suggested by Ajzen and Fishbein [47]. Two questions, based on suggestions by Ajzen and Fishbein [47], measured the affective component (i.e., drives, emotions) of performing a behavior [48] and assessed affective, salient, behavioral beliefs (i.e., factors the individual would like and dislike about engaging in the behavior). One question was designed to capture any additional behavioral beliefs the participant had about enrolling in the online intervention. The remainder of the questions were used by Ajzen and Driver [49]. Four open-ended questions measured normative beliefs (i.e., individuals or groups likely to approve and disapprove of the behavior; individuals or groups most likely and least likely to engage in the behavior). Two open-ended questions assessed control beliefs (i.e., factors that would enable and prevent them from engaging in the behavior). One additional question was simply added to check the attention of the participants. All items reflected the expectancy components of behavioral beliefs rather than expectancy x value combinations. According to the TPB, behavioral beliefs should be weighted by the value attached to the outcome. However, assessing expectancy alone not only reduces participant burden and eliminates scaling and analysis problems, but also strengthens correlations with TPB constructs [50,51]. This approach, with the same items, has been used by other investigators conducting studies on health promotion [50,52].

### 2.3. Data Analysis

Descriptive statistics were calculated to characterize the sample using SPSS version 22. Two authors (i.e., KR and CM) conducted a content analysis using emergent coding according to the recommended guidelines to determine the underlying themes [53]. First, the two raters independently reviewed the data. The primary coder (i.e., KR) made a list of key words and content that emerged through the data and created broader categories based on concepts of similar meaning (i.e., content categories). Next, the raters compared the content categories to the original data and reconciled differences in codes, creating a consolidated list of content categories. Third, the raters used the list of agreed-upon content categories to independently identify the number of times the content categories appeared in the text for two randomly selected participants. Finally, the raters independently determined the frequency of occurrence of each category in the two randomly selected participants. The reliability of coding was deemed acceptable once discrepancies were discussed and mutually agreed upon. A 100% agreement rate between raters was reached across all codes, which exceeded the recommended 95% reliability [53]. The agreement rate was determined by adding up the number of coding agreements and then dividing by the total number of codes. The content categories were listed in order of frequency of occurrence, with more frequently occurring content categories indicating a higher level of significance [54].

## 3. Results

### 3.1. Salient Beliefs

The content analysis resulted in 19 content categories across behavioral beliefs, normative beliefs, and control beliefs. There were 191 supporting text references.

#### 3.1.1. Behavioral Beliefs

Nine behavioral beliefs emerged through the content analysis with a total of eighty-two supporting references in the text. The strongest category to emerge was *access and convenience*, with a total of 21 text references. These references commonly included access to materials and professionals, convenience, and flexibility. *Access and convenience* was viewed primarily as a facilitator (i.e., seen as an advantage or likeable). For example, a typical response was mentioned by a 52-year-old married mother of a teenage girl with ADHD, anxiety, and depression and who was employed full-time: “*It would be easy to access. I feel like sometimes I don’t make as many appointments as I could because the time and place are inconvenient*.” (Question 1 Advantage; Participant 4). A representative minority view came from a 35-year-old married mother of a girl with ASD and who was also employed full-time. She indicated feeling worried about the “*Lack of flexibility*” (Question 2 Disadvantage; Participant 17) that may result from the intervention being delivered in an online format.

*Social support* was identified as the next strongest behavioral belief to emerge, with 18 supporting text references emerging from both male and female parents and caregivers; quotes typically emphasized social support from peer parents and lack of one-on-one attention. Thus, *social support* was considered both a facilitator and barrier, respectively. For example, a typical response was mentioned by a 40-year-old mother of a teenage girl with ADHD, conduct disorder, and oppositional defiant disorder and who reported being in a domestic partnership and being employed full-time: “*It helps me find support in other parents who are also needing support with regard to issues their child is having*...” (Question 1 Advantage; Participant 7). Another typical response came from the 35-year-old married mother of a girl with ASD, who was employed full-time, and demonstrated concern about the type of social support. Specifically, she was worried that there would be “*No face-to-face interaction*” (Question 4 Dislike; Participant 17) given the program’s online platform.

*Effectiveness* was another important behavioral belief which was identified, with eight text references emphasizing the importance of having an improved chance of success and efficient services. *Effectiveness* was expressed as being primarily a barrier. For example, one typical quote came from a 29-year-old married stepfather of a teenage boy with anxiety, bipolar disorder, and a specific learning disorder and who expressed concern about program effectiveness: “*I would not dislike anything about enrolling, unless services are not beneficial*…” (Question 4 Dislike; Participant 11). Alternatively, the same participant also expressed a representative minority view that such a program would *“give me a better chance at success”* (Question 1 Advantage; Participant 11).

*Skill development* was another important behavioral belief, with eight text references. *Skill development* was expressed as being solely a facilitator. For example, one typical response came from the 29-year-old married stepfather of a teenage boy with anxiety, bipolar disorder, and a specific learning disorder who was also employed full-time: “*I may learn things as a young parent that others already know and can guide me with*” (Question 3 Like; Participant 11). Another representative quote was taken from a 45-year-old low SES married father of a teenage boy with anxiety, ASD, and bipolar disorder, and who was employed full-time. He expressed the desire to “…*learn the skills needed to care for my child*” (Advantage; Participant 12).

Another behavioral belief was regarding *cost*, with seven supporting text references among middle and upper SES respondents. *Cost* was expressed as being primarily a barrier. For example, a 60-year-old married mother of a boy with ASD and who was employed full-time indicated there would be “*No disadvantages, except maybe cost*” (Question 2 Disadvantage; Participant 2). A representative minority view regarding cost was expressed by the 45-year-old married mother of a boy with ASD and a specific learning disorder who was employed part-time. She indicated the program “…*might also be cheaper than the alternatives*” (Like; Participant 15).

*Parent burden* was another behavioral belief that was identified, with seven text references revolving around parenting stress and the relief associated with enrolling in the intervention that was expressed by both men and women. *Parent burden* was expressed as being both a facilitator and barrier. For example, one representative quote came from the 35-year-old married mother of a girl with ASD, and who was employed full-time. She indicated the program afforded the opportunity to “*Reduce stress*” (Question 1 Advantage; Participant 17). Another typical response was expressed by a 33-year-old mother of a boy with ADHD, ASD, and a specific learning disorder who reported being in a domestic partnership and being employed full-time. She was concerned about the potential for increased parent burden, saying “*The time it takes may be more overwhelming*” (Question 4 Dislike; Participant 1).

Another behavioral, regarding *privacy*, included five text references revolving around confidentiality with private information. *Privacy* was expressed as being both a facilitator and barrier. For example, a 46-year-old married mother of a teenage girl with anxiety and depression who was employed full-time mentioned, “*I’d like the anonymity of it. Also, I don’t tell a lot of people about this*.” (Question 3 Like; Participant 5). A 44-year-old low SES divorced mother of a teenage girl with anxiety, bipolar disorder, and oppositional defiant disorder, and who was unemployed not by choice, reported a potential barrier being: “*The only thing I can imagine would be having privacy concerns*” (Question 4 Dislike; Participant 9).

*Improved relationship and interactions with youth* was also expressed as a behavioral belief with a total of four text references across men and women respondents. *Improved relationship and interactions with youth* was identified as being solely a facilitator. For example, a typical response was exemplified by the 40-year-old mother of a teenage girl with ADHD, conduct disorder, and oppositional defiant disorder, and who reported being in a domestic partnership and being employed full-time. She said, “*I love that it enables me to interact more positively with my daughter, which addresses both her behavior and our relationship with each other*” (Question 3 Like; Participant 7).

The final behavioral belief identified was *increased parent engagement in promoting health behaviors in youth*, with a total of four text references supporting its status as a facilitator. For example, a 46-year-old divorced father of a teenage girl with anxiety, ASD, and depression who was employed full-time reported: “*I think it would get me thinking more about making an effort to help my kid lose weight and be more active*” (Question 1 Advantage; Participant 20).

#### 3.1.2. Normative Beliefs

Five normative beliefs emerged through the content analysis with a total of 58 supporting references in the text. The strongest category to emerge was *family (e.g., spouse, grandparents, siblings, children)* with a total of 18 supporting text references. *Family* was listed as a facilitator (thought others would approve, thought others would be most likely to enroll) and barrier (thought others would disapprove, thought others would be least likely to enroll). For example, a typical response was mentioned by a 45-year-old employed father of a boy with anxiety and depression, who in response to the question of who would approve of enrolling in this intervention said, “*My wife, my family, my child*” (Question 5 Approve; Participant 3). Alternatively, family was also mentioned as a potential barrier by a 29-year-old employed stepfather of a teenage boy with anxiety, bipolar disorder, and a specific learning disorder who said, “*My grandfather, [name excluded]. He is very insular and does not like to socialize*” (Question 6 Disapprove, Participant 11).

*Parents and caregivers* was identified as the next strongest normative belief category to emerge, with 16 supporting text references. *Parents and caregivers* similarly was both a facilitator and barrier. Typical responses included a quote from a 44-year-old low-income, divorced mother of a teenage girl with anxiety, bipolar disorder, and oppositional defiant disorder and who, in response to who would be most likely to enroll, reported: “*I think anyone who is a parent, sibling or family member of someone who has MI would be likely. Having access to any resource that could help them be supportive seems like it would be attractive to all sorts of people*” (Question 7 Most likely; Participant 9). Alternatively, a 45-year-old part-time employed mother of a boy with ASD and specific learning disorder said, “*Guardians might be apprehensive about inefficient [ineffective] services*” (Question 6 Disapprove; Participant 15).

*Medical professionals* was another important normative belief category that was identified. There were 10 text references which included doctors, pediatricians, physical therapists, and occupational therapists. The *Medical professionals* category was expressed as being both a facilitator and barrier. For example, a 40-year-old employed mother of a teenage girl with ADHD, conduct disorder, and oppositional defiant disorder indicated their “*Pediatrician or primary care doctor, physical and occupational therapists*…” (Question 5 Approve; Participant 7) would approve. Meanwhile, some participants, such as a 33-year-old employed mother of a boy with ADHD, ASD, and a specific learning disorder, were worried “*His doctor*” (Question 6 Disapprove; Participant 1) would disapprove.

Another important normative belief was *school staff*, with seven supporting text references, including teachers and principals. *School staff* was also expressed as being both a facilitator and barrier. Typical responses included a 33-year-old employed mother of a boy with ADHD, ASD, and a specific learning disorder who said “…*his school*” (Question 5 Approve; Participant 1) would approve. Another representative example includes a 49-year-old single employed stepfather of a boy with an emotional and behavioral disorder and schizophrenia who indicated school staff including “*Principles*...” (Question 8 Least likely; Participant 8) would be the least likely individuals to approve of enrollment in such a program.

The final normative belief to emerge was the category of *mental health professionals*, which included psychiatrists, social workers, and counselors and was supported by seven text references. *Mental health professionals* also was identified as both a facilitator and barrier. For example, one typical response came from a 40-year-old employed mother of a teenage girl with ADHD, conduct disorder, and oppositional defiant disorder who reported “…*mental health provider*” (Question 5 Approve; Participant 7) as an individual who would approve of her enrollment. Another representative response came from a 49-year-old single employed stepfather of a boy with an emotional and behavioral disorder and schizophrenia who reported “...*psychiatrist*” (Question 8 Least likely; Participant 8) as an individual who was least likely to approve of enrolling in the program.

#### 3.1.3. Control Beliefs

Five control beliefs emerged through the content analysis and had a total of 51 supporting references in the text. The strongest category to emerge was *cost,* with a total of 21 supporting text references. *Cost* was both a facilitator (would make it easy to enroll) and barrier (would make it difficult to enroll). For example, one representative response came from a 60-year-old employed mother of a boy with ASD who said, “*Affordable cost*…” (Question 10 Easy; Participant 2) would make it easy to enroll in the program. Another typical response came from a 33-year-old employed mother of a boy with ADHD, ASD, and a specific learning disorder who indicated “*Having to pay a lot of money for this service*…” (Question 11 Difficult; Participant 1) would make it difficult to enroll in the program.

The second most salient control belief was *access and convenience*, with a total of 12 text references which indicated the importance of convenience and access to materials necessary for enrolling in the program (e.g., internet and computer access). *Access and convenience* was referenced as a facilitator and barrier. One representative quote came from a 29-year-old employed stepfather of a teenage boy with anxiety, bipolar disorder, and a specific learning disorder, who said, “*A employed laptop or computer, a way to access this online intervention through a smart phone and fast internet access*” (Question 10 Easy; Participant 11) would enable his enrollment in the online intervention. In reference to what would make enrollment difficult, a 44-year-old low-income divorced mother of a teenage girl with anxiety, bipolar disorder, and oppositional defiant disorder said, “*No access to a computer or reliable internet service*” (Question 11 Difficult; Participant 9).

Another important control belief was *easy enrollment*, with 10 supporting text references. *Easy enrollment* was both a facilitator and barrier. For example, a typical response was mentioned by a 52-year-old employed mother of a 16-year-old daughter with ADHD, anxiety, and depression who said a factor that would make enrollment easy would be to “*Have an easy enrollment process*” (Question 10 Easy; Participant 4). Conversely, when asked about what would make enrollment difficult, a 46-year-old employed mother of a teenage girl with anxiety and depression said “…*difficulty getting signed up, a lot of wait time to be approved*” (Question 11 Difficult; Participant 5).

*Intuitive (e.g., layout, platform, detailed instructions)* was another important control belief that emerged with the support of five text references, highlighting the importance of having the intervention on a platform that would be easy to use. *Intuitive* was solely identified as a facilitator. One typical response was mentioned by a 45-year-old part-time employed mother of a boy with ASD and a specific learning disorder who said, an “…*intuitive platform*…” (Question 10 Easy; Participant 15) would make enrollment easier. A 36-year-old employed father of a boy with anxiety produced a similar response, saying “…*website layout*” (Question 10 Easy; Participant 10) would influence the ease of enrollment.

The final control belief to emerge was *privacy,* with support from three text references that emphasized the importance of a secure site and HIPAA compliance. *Privacy* was identified as a facilitator and barrier. For example, a 40-year-old employed mother of a teenage girl with ADHD, conduct disorder, and oppositional defiant disorder indicated a “…*secure site, HIPAA compliance*” (Question 10 Easy; Participant 14) would enable her enrollment in the program. Alternatively, privacy was also identified as a factor that could prevent enrollment. One such belief emerged from the 40-year-old mother of a teenage girl with ADHD, conduct disorder, and oppositional defiant disorder, and who reported being in a domestic partnership and being employed full-time. She said, “…*data being sold to third parties, web security*” (Question 11 Difficult; Participant 7) would be a preventative factor in her decision to enroll.

## 4. Discussion

The current study is among the first to examine the salient beliefs associated with parent intention to enroll in an educational program to support the development of parents’ and guardians’ skills to support the healthy lifestyle behaviors of youth with OW/OB who take psychotropic medication. The results of this study revealed potentially important beliefs (i.e., behavioral beliefs, control beliefs, normative beliefs) which likely influence theoretical attitudinal constructs related to parents’ intentions to enroll in such a program. Several important salient beliefs common among parents of youths with OW/OB who take psychotropic medication were identified, including behavioral beliefs such as the need for social support and engagement with similar families; normative beliefs such as influential family members, school personnel, and medical and mental health providers; control beliefs such as easy enrollment and intuitive design; and combined beliefs such as cost, access and convenience, privacy and anonymity. Investigators often incorrectly assume that direct measures of theory of planned behavior constructs are obtained by asking arbitrarily selected questions or by adapting items that were included in previous studies. However, Ajzen [29] noted that this approach may yield findings of interest, but that it produces measures with relatively low reliabilities and leads to underestimating the relationships between the theory’s constructs and the theory’s predictive validity. Ajzen [29] emphasized that it is necessary to select appropriate items through formative stages of investigation, such as the design of the present study. The themes identified through this formative work may be used in a future study to inform the development of a quantitative measure of salient beliefs and measure their association to attitudinal constructs and intention as outlined by the theory of planned behavior. As a secondary goal, these themes provide useful preliminary insights regarding potentially important considerations for the development of a future online parent intervention.

One clear theme, which was found among the salient behavioral beliefs, was that the opportunity to receive social support would be a substantial advantage to enrollment in an online health promotion intervention. Specifically, participants clearly stated a desire for social support and indicated a preference for communicating with other parents with similar parenting concerns. This theme was echoed in the findings of several other studies of parents of youths with a variety of health conditions, such as cystic fibrosis [55], chronic kidney disease [56], and ASD [57]. Other work has shown the positive outcomes associated with receiving social support. Specifically, several studies have found that perceived social support from others is associated with significantly reduced parental stress and self-stigma and significantly improved psychological well-being among individuals in these parenting roles [58,59,60]. Notably, among parents, informal social support from friends fostered greater protection against stress [58] and self-stigma [60] than support provided by professionals or family members. Similar psychosocial benefits of perceived social support have been found among parents of adults with MHD [61]. In the current study, parents and guardians primarily referred to receiving informational support and benefiting from social learning through stories and tips shared by other families.

Additional themes found among the salient normative beliefs were that family members and professionals (i.e., medical, mental health, schools) would be influential to parent enrollment in an online health promotion intervention. Participants reflected that family members and professionals may either support enrollment, and be likely to enroll in such a program themselves, or be viewed as unsupportive. Related literature highlights the importance of incorporating these groups into interventions for parents and guardians of youth with MHD. For example, parents of youth with ASD have expressed a desire for online interventions to be more widely accessible to family members and professionals (e.g., the provision of psychoeducation and printable resources for supportive others, the incorporation of healthcare professionals into the online community) [57]. In addition to influencing enrollment, family can also be a significant source of support in promoting and modeling healthy lifestyle behaviors to youth with OW/OB and MHD [25]. Assessing the importance of extended family member engagement may also be an important cultural consideration, bolstering the protective value of family unity and cohesion [62,63,64]. Professionals could be influential in having conversations with family units that are focused on health promotion (i.e., promoting a healthy diet and activity patterns) rather than weight-focused discussions, which may have unintended consequences (e.g., unhealthy weight-control behaviors, weight gain, eating disorders, and greater body dissatisfaction) [65,66]. Additionally, professionals could serve vital roles as referral sources for online interventions for this population. Specifically, interventions could increase access and engagement through developing partnerships with providers and schools and asking these professionals to personally encourage enrollment and distribute marketing material to parents [67,68]. Partnered providers could be advised to speak with parents about whether there are other key family members they would like to invite to enroll and who may also benefit from accessing resources from an online healthy lifestyle intervention.

While the need for peer and provider support is common among studies, some participants in this study mentioned concerns about privacy and desire for anonymity. This theme was found among both behavioral beliefs and control beliefs. These concerns were echoed in related literature, in which parents highlighted the importance of online interventions implementing security agreements and creating private profiles with unidentifiable information [57]. Therefore, close attention to the types of support emphasized and format and methods for providing support to this population may be particularly important. Online social support opportunities may be offered in a variety of formats. This could involve synchronous communication among users in which there is live interaction with other individuals, a group, or experts (e.g., live drop-in appointments, online scheduled appointments) [57]. The most common format is asynchronous communication, where members may read and respond to messages or post messages at the members’ convenience (e.g., searchable discussion boards which can be filtered by topic or disorder) [55,57,69]. However, parents have expressed their desire for online interventions to include both synchronous and asynchronous components [57]. Furthermore, perceptions of presence and reciprocity in communication, either synchronous or asynchronous, have proven valuable in influencing participation and retention in online support programs [70]. Additionally, the trustworthiness of online interventions for parents may be enhanced if the site demonstrates provider support through the inclusion of an “ask the expert” or “frequently asked questions” feature, or a resource hub with links to local providers and credible supports [57,71,72], thus bolstering parent participation and retention in such a program [70]. Some similar themes were echoed in a qualitative study of a tool to support family caregivers (e.g., a patient-oriented digital decision-making solution: a doctor-at-home system). Zippel-Schultz [73] and colleagues, showed that important tool features such as ongoing reassurance and support to manage heart failure and trust in the technology (e.g., it has been developed by experts) are important in determining the acceptance of the technology [73]. Taken together, it is likely that an online healthy lifestyle intervention for parents of youths with MHD may benefit from the inclusion of peer and expert supports delivered in both synchronous and asynchronous formats in a manner that respects parent anonymity. However, additional investigation is required to confirm this hypothesis.

Areas identified by parents as influencing their decision to enroll in online parenting interventions, which were found among the salient control beliefs, were easy enrollment and the intuitive design of the online intervention (i.e., ease of site navigation, detailed instructions). These results are consistent with one of the two key theoretical constructs in the TAM model [39]. Once a technology has been developed, features related to ease of use would be expected to predict intentions to use the tool. This preference has appeared in other work where important online intervention features included being able to navigate a site easily, having easily understandable language, and having operational links to other sites [74]. Specifically, participants indicated a preference for an easy-to-navigate landing page with a clear enrollment icon as an important feature. Future research could examine the relationship between salient beliefs regarding ease of use and the development of attitudes toward enrolling in the intervention, as well as intention to enroll in the program. In addition, work to understand the acceptability of a preliminary online intervention website for this population would help to identify specific features of the platform that are associated with the perception of clarity and ease of navigation. Iterative adjustments could then be made based on direct feedback from end-users, and acceptability regarding clarity for enrollment and ease of navigation re-examined.

Additionally, themes regarding cost, access, and convenience were also identified as salient control beliefs and behavioral beliefs in this population of parents. Specifically, parents referenced cost as a crucial factor which could either impede or bolster their desire and ability to enroll in an online healthy lifestyle intervention. This finding has been echoed in another study of caregivers for adults with serious mental illness which was conducted in India [75]. Other work has shown that the digital delivery of an intervention is hypothesized to be a cost-effective alternative to traditional face-to-face delivery, increasing both program sustainability and accessibility for parents [76,77,78]. However, more research is needed on the cost-effectiveness of internet interventions, which could then be used to support policy changes [79]. Participation and retention in such an intervention may also be improved by parents being able to access relevant information for supporting their youth and by being able to access a program that is flexible (e.g., flexibility in message, modality, and delivery) [70]. In the current study, parents noted the importance of an intervention being flexible and being able to access the intervention on their phones or having access to a computer and reliable internet. Intervention developers can improve intervention accessibility by ensuring the platform is supported for use with computers and phones, and by providing a list of local resources parents could use if they do not have personal access to a computer or internet (e.g., a public library).

### 4.1. Limitations

These results should be interpreted within the context of the study’s limitations. First, parents and guardians were self-selected for participation through convenience sampling on MTurk, which could bias results based on participant’s reasons for participation and limit the generalizability to the population of interest. Second, while low-quality responses were filtered during screening, responses to the open-ended questionnaire were briefer and provided more limited context compared to traditional face-to-face interviews. Third, these results may not generalize to similar populations who may benefit from interventions, such as parents and guardians of youth with OW/OB who have a mental health disorder but do not take psychotropic medication or youth who take a psychotropic medication and have experienced significant weight gain but do not yet meet criteria for OW/OB. Fourth, clinical history information was collected based on parent and guardian self-reporting, which may be susceptible to error and bias. Finally, this study was conducted prior to COVID-19 which may have altered factors influencing parent intention to enroll in such a program (e.g., parents may now be even more willing to enroll in an online program).

### 4.2. Future Directions

Future research should focus on developing a quantitative salient belief questionnaire based on data obtained from the elicitation of salient beliefs. Psychometric properties of such a measure (e.g., internal consistency, test–retest reliability, validity) could be examined. Still, other work should investigate the relationship between salient beliefs and the attitudinal theory of planned behavior constructs and intentions of parents and guardians to enroll in an online intervention to develop behavioral health coaching skills to support healthy lifestyles among their youths with MHD and OW/OB. Future work may also include a focus on general participant attitudes toward participating in an online intervention of the nature described in this study, as well as examine differences in participant characteristics between those with more and less favorable attitudes. This research represents a focus on parent-oriented programs. However, youths aged 11–17 would benefit from the development of a parallel online program directly addressing their unique health promotion needs and preferences. Future formative research should be conducted to understand salient beliefs of youths regarding participation in such programs as well as how these beliefs and attitudinal constructs within the TPB are associated with intention to enroll in a program. Once a tool is created, future work in both parent/guardian and youth samples could examine factors associated with the decision to adopt or use these programs.

## 5. Conclusions

OW/OB frequently impacts youths who take psychotropic medications, yet few interventions have been tailored to the needs of these youths or their parents and guardians. In the current qualitative elicitation study, several salient beliefs that are consistent with the TPB were identified as being influential to parent enrollment in an online healthy lifestyle intervention for their youth. Intervention developers should consider concentrating efforts toward the program’s intuitive design; ease of enrollment; affordability; the privacy and anonymity of end-users; the social support opportunities offered; and the incorporation of influential family members and professionals into different stages of the intervention (e.g., initial enrollment, active participation).

## Figures and Tables

**Table 1:** Parent and guardian demographics (*n* = 20).

Characteristic	M ± SD (Range)	*n* (%)
Age	42.5 ± 7.8 (29–60 years)	
Cisgender Female ^a^		11 (55.0)
Race ^b^		
White		17 (85.0)
American Indian/Alaska Native		3 (15.0)
Black		3 (15.0)
Latinx/Hispanic (Non-White)		1 (5.0)
Ethnicity		
Not Hispanic/Latino		18 (90.0)
Hispanic/Latino		2 (10.0)
Born in the United States of America		18 (90.0)
Marital Status		
Married		14 (70.0)
Domestic Partnership		3 (15.0)
Divorced		2 (10.0)
Single		1 (5.0)
Education Level		
Some College/Associate’s Degree		7 (35.0)
Bachelor’s Degree		11 (55.0)
Some Graduate School/Postgraduate		2 (10.0)
Employment Status		
Full-time		17 (85.0)
Part-time		1 (5.0)
Unemployed		2 (10.0)
Net Income		
USD 50,001 and above		12 (60.0)
USD 25,001–50,000		6 (30.0)
Less than USD 25,000		2 (10.0)
Relationship to Youth		
Parent		16 (80.0)
Stepparent		3 (15.0)
Foster parent		1 (5.0)

^a^ All participants (*n* = 20) identified as cisgender. ^b^ Three participants identified as multiracial.

**Table 2:** Salient Belief Elicitation Questionnaire.

Instructions: Please take a few minutes to tell us what you think about the possibility of enrolling in an online health and wellness intervention for your child or adolescent with a mental health disorder. The program would be an online course that included reading material, watching videos, and engaging in supportive interactions with other parents that would teach you to be your child’s behavioral coach. Your child or adolescent would be encouraged to engage in a new way of eating and increase their physical activity. The time involved weekly may vary depending upon the content involved and the outside activities in which you choose to engage. Please list whatever thoughts come freely to your mind with the understanding that there are no right or wrong answers, we are just interested in your personal opinions.
What do you believe are the advantages of enrolling in an online health and wellness intervention in order to help your child or adolescent with a mental health disorder?
2. What do you believe are the disadvantages of enrolling […]?
3. What do you believe you would like about enrolling […]?
4. What do you believe you would dislike about enrolling […]?
5. Please list the individuals or groups who would approve or think you should enroll […]?
6. Please list the individuals or groups who would disapprove or think you should not enroll […]?
7. Sometimes, when we are not sure what to do, we look to see what others are doing. Please list the individuals or groups who are most likely to enroll […]?
8. Please list the individuals or groups who are least likely to enroll […]?
9. Please write “I am putting forth my best effort” to indicate you are still paying attention.
10. Please list any factors or circumstances that would make it easy or enable you to enroll […]?
11. Please list any factors or circumstances that would make it difficult or prevent you from enrolling […]?
12. What else comes to mind when you think about enrolling […]?

Note: All items ended in the same wording (i.e., in an online health and wellness intervention in order to help your child or adolescent with a mental health disorder). Participants who initially did not indicate they had a youth with a mental health disorder received a similar prompt and items that included the term “youth” in place of “child or adolescent with a mental health disorder.”

## Data Availability

De-identified data will be made available upon reasonable request to corresponding author.
